# Norm mining, identification, and detection: a systematic literature review

**DOI:** 10.3389/frai.2026.1702659

**Published:** 2026-02-26

**Authors:** Benoît Alcaraz, Yazan Mualla, Sukriti Bhattacharya, Igor Tchappi, Vincent de Wit, Amro Najjar

**Affiliations:** 1 Department of Computer Science, University of Luxembourg, Esch-sur-Alzette, Luxembourg; 2 Université de Technologie de Belfort Montbéliard, Belfort, France; 3 Luxembourg Institute of Science and Technology, Esch-Belval, Luxembourg

**Keywords:** data mining, norm identification, normative systems, norms, systematic literature review

## Abstract

This paper presents a systematic literature review on norm identification in multi-agent systems. Norms play a crucial role in guiding agent behavior, ensuring cooperation, and resolving conflicts. By analyzing 35 selected studies, we categorize methods for detecting, synthesizing, and adapting norms in multi-agent systems. We also examine their effectiveness in dynamic and uncertain environments. The findings highlight gaps in current approaches, including scalability, adaptability, and real-world applicability. Future directions emphasize the integration of Large Language Models, testing in complex environments, and fostering interdisciplinary collaboration to advance socially aware autonomous systems.

## 1 Introduction

Autonomous agents have rapidly moved from theoretical constructs to real-world applications. Notable examples include autonomous cars, intelligent personal assistants, and smart factory mobile robots (Vamvakas et al., 2023; Zhu and Zhao, 2021; Yu et al., 2021; Nguyen et al., 2020). AI-powered assistants, drones, and robotic systems are increasingly integrated into human-centric environments, where they must adhere to expected social and regulatory norms. Ensuring that these autonomous agents behave appropriately in diverse and dynamic settings is a critical challenge.

In human societies, norms serve as implicit or explicit guidelines that govern interactions, ensuring coordination, safety, and efficiency (Morris et al., 2015; Pham et al., 2024). These norms can be legal (e.g., traffic laws for autonomous vehicles), social (e.g., queuing behavior in public spaces), or ethical (e.g., fairness in AI decision-making). These norms can sometimes be made explicit through text, but can also emerge dynamically through repeated interactions and may vary across cultural and situational contexts (Mc Breen et al., 2011). They can also change and evolve (i.e., *norm drifting*) (Boella et al., 2009). Therefore, manually specifying all possible norms for autonomous agents is not a viable solution for large and changing environments. Instead, agents must be equipped with the capability to autonomously identify, learn, and adapt to norms. This process, known as norm identification or norm mining, involves the automatic detection, extraction, and validation of norms based on observed behaviors, interactions, communication, or analysis of external regulatory sources.

Over the past fifteen years, efforts have been devoted to developing methods for norm identification. These approaches aim to enable autonomous agents to recognize both explicit and implicit norms (as sometimes, norms can be communicated, but can also be embedded into the behavior of some agents without being written or represented somewhere), reason about their applicability, and adjust their behavior accordingly. Usually, the main goal of those approaches is to translate the extracted norms into a form matching the Deontic Logic representation of the norms. In Deontic Logic, regulative norms can be categorized in three modalities, namely Prohibitions, Permissions, and Obligations (Gabbay et al., 2021). Using those modalities then allows for an agent to reason to know whether its behavior is complying or not with the norms of the system.

This paper provides a systematic literature review (SLR) of the research in norm identification, also referred to as norm detection, or norm mining, over the last fifteen years (i.e., 2009 to 2024). We categorize existing approaches and highlight open challenges and future research directions. By synthesizing advancements in this field, we aim to contribute to the development of more socially aware and norm-compliant autonomous agents capable of seamlessly integrating into human environments. Our main motivation for this contribution is that the only survey (Savarimuthu, 2011) we are aware of was published in 2011. Thus, it misses many recent works that have been done in the area.

First, we introduce in Section 2 the methodology used to collect and classify the papers. We also introduce a range of research questions that this paper aims to answer. Then, in Section 3, we analyze the collected papers both quantitatively and qualitatively under different aspects. In Section 4, we identify several challenges and research directions for future work in the area of norm identification. Finally, we conclude and provide some insights, as well as a roadmap for future work, in Section 5.

## 2 Methodology and objectives

In the last decade, research on computer science in general and artificial intelligence in particular has witnessed a significant increase both qualitatively and quantitatively. For this reason, SLRs are becoming popular to help analyze the evolution of these domains. Kitchenham and Charters (2007) define the SLR as follows: “A form of secondary study that uses a well-defined methodology to identify, analyze, and interpret all available evidence related to a specific research question in a way that is unbiased and (to a degree) repeatable.” Where secondary study refers to “a study that reviews all the primary studies relating to a specific research question.”

**Figure 1:**
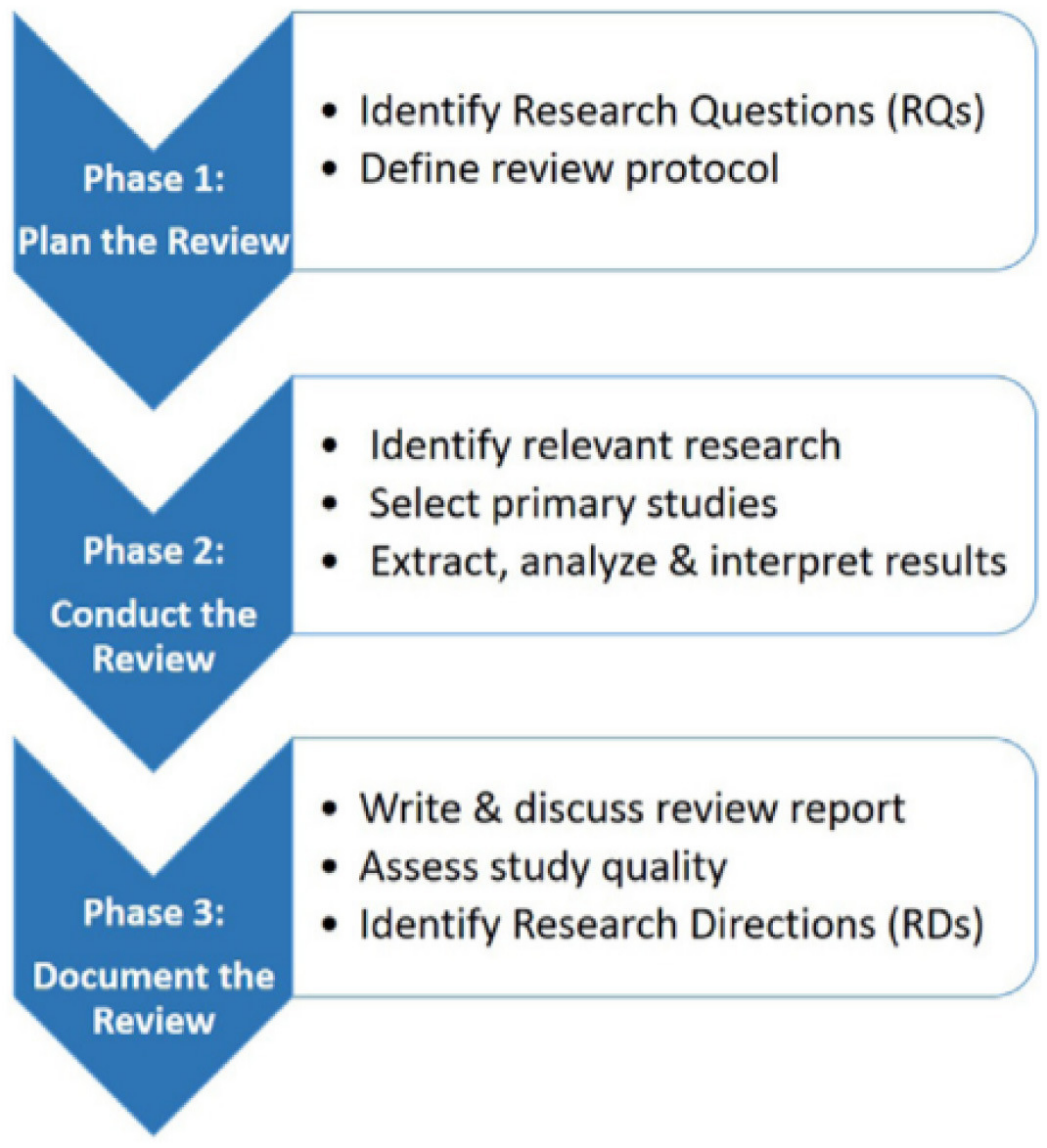
The systematic literature review process, adapted from Brereton et al. (2007), Mualla et al. (2019), and Galster et al. (2013).

In this paper, we define a primary study as a research paper addressing a specific research question in the domain of *Norm Identification*, or *Norm Mining*. The aim of SLRs can be threefold (Kitchenham and Charters, 2007): (i) to summarize the existing evidence concerning a specific technology that is being used broadly, (ii) to identify gaps in the existing research to suggest areas for future investigation, and (iii) to provide a background allowing to position new research activities. With these goals in mind, we base our SLR on Budgen and Brereton (2006) and Kitchenham et al. (2010), which are among the most common methodologies for computer science SLRs. Such an approach ensures rigorousness, fairness, and reproducibility. Figure 1 illustrates the review process.

This section is organized as follows. First, Section 2.1 highlights the research questions. Second, Section 2.2 explains the review protocol, how conflicts are resolved, and how biases are overcome. Third, in Section 2.3, the defined protocol is executed and the review process is undertaken (document collection, conflict resolution, etc.).

### 2.1 Research questions

To systematically explore this domain, several key research questions have been formulated. These questions aim to address critical aspects of normative systems, ranging from detection and identification to synthesis and adaptation within multi-agent systems (MAS). This section elaborates on these research questions, providing hints on their significance and the assumptions underlying them.

**–RQ.1:** How to identify the norms of a society by observing it?—This question focuses on passive observation techniques that allow agents to detect prevailing norms without direct interaction.
**–RQ.2:** How to identify the norms of a society by interacting with it?—While passive observation provides valuable insights, active interaction offers additional dimensions for norm identification.
**–RQ.3:** How to differentiate individual norms from societal norms in a multi-agent society?—Differentiating between individual and societal norms is crucial for understanding the emergence and enforcement of normative behavior. Sub-questions RQ.3.1 and RQ.3.2 further explore the feasibility of distinguishing personal norms (p-norms) from group norms (g-norms) and the methodological challenges involved.

**–RQ.3.1:** Is it possible to differentiate a p-norm from a g-norm?
**–RQ.3.2:** How to perform this differentiation with only one agent?

**–RQ.4:** How to detect prohibition norms without norm enforcement?—Detecting prohibition norms in the absence of explicit enforcement mechanisms is a challenging task.
**–RQ.5:** How to detect norms in communications?—Communication analysis offers a rich source of normative information. Natural language processing (NLP) techniques play a crucial role in extracting norms from textual interactions.
**–RQ.6:** Is it possible to adapt to drifting norms without restarting the whole learning phase?—Adapting to norm drift without reinitializing the learning process is essential for maintaining normative coherence in evolving societies. Norm evolution presents significant challenges for adaptive agents.
**–RQ.7:** Is it possible to detect sub-communities of agents?—The emergence of sub-communities within a multi-agent society can indicate norm fragmentation.

### 2.2 Review protocol

**Figure 2:**
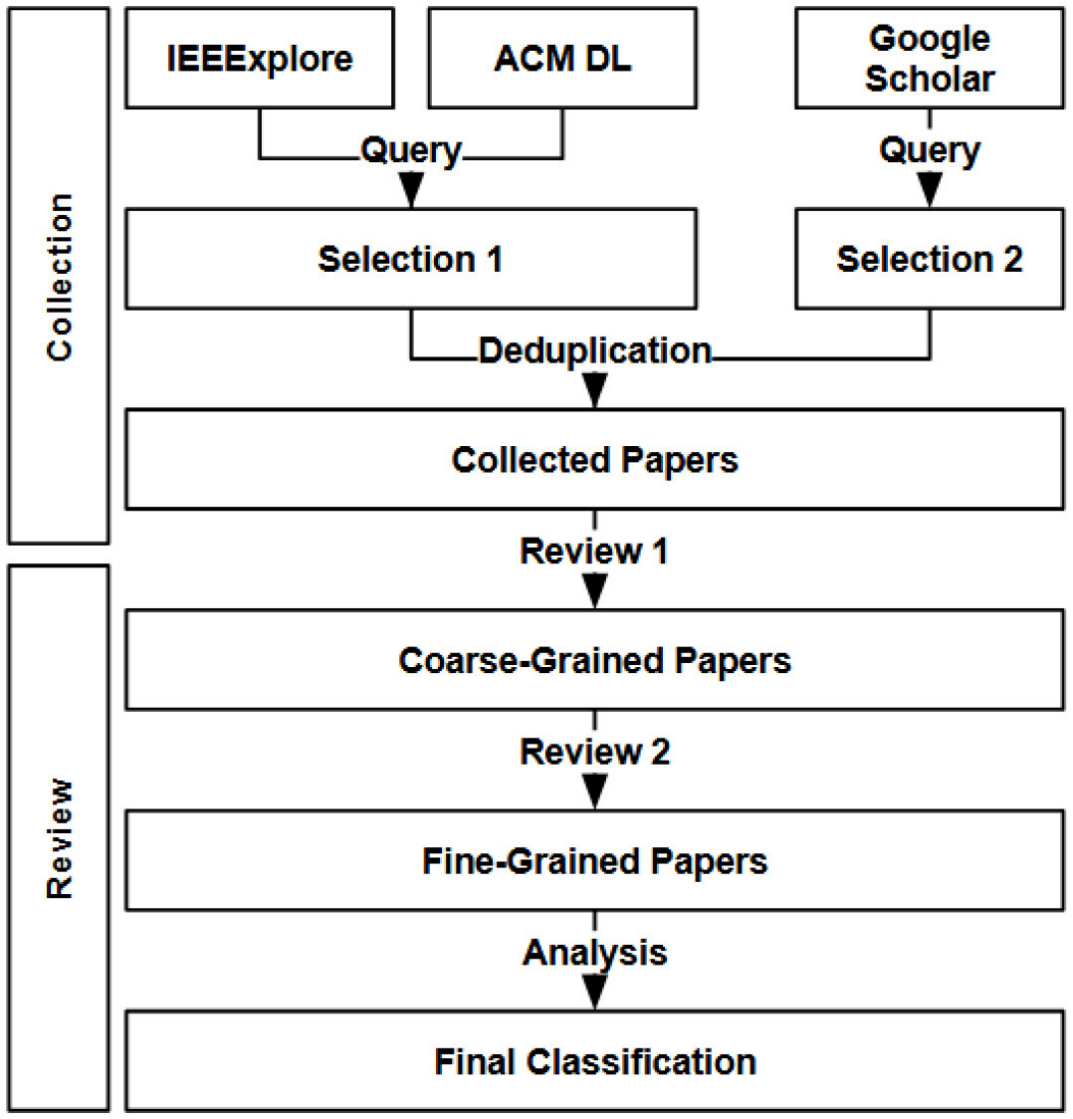
Flow of the followed process from the gathering of the papers to the final results.

This section describes in detail the guidelines we follow for our review, as well as the execution of the process, indicating how many papers were included or excluded, and on which criterion it was based. An overall view of the whole process is shown in Figure 2.

#### 2.2.1 Database selection

This process is composed of the following couple of steps:

(a) IEEExplore, ACM Digital Library, and Google Scholar are selected as the three databases constituting the source of information. The selection of the first two databases is prominent in computer science. Google Scholar is selected because it provides a large list of documents that are not indexed in the two previous databases, e.g., papers from conference proceedings.
(b) The databases are queried with a set of keywords. These keywords are devised based on the authors' knowledge of the norm mining domain.

When queried with these keywords, each database responded with a set of articles that were considered by the reviewing process. The number of articles to be produced by the queries is relatively large for IEEExplore, ACM DL, and Google Scholar databases. However, only a few of these articles were relevant to the research questions raised in the previous section. For this reason, as in Calvaresi et al. (2017), the following stop criterion was applied: “Stop the collection of articles after a sequence of 15 titles, completely incoherent with the query, appeared in the list.” Determining whether an article is coherent is left to the reviewers' subjective view when they deem that there was no adherence between the query performed on the database and the title or abstract of the article appearing in the result.

##### Supplementary retrieval strategy

In addition to keyword-based queries on IEEE Xplore, ACM Digital Library, and Google Scholar, we conducted a light citation-based screening during the fine-grained selection phase. Specifically, references of included papers were inspected to identify potentially relevant works not retrieved by the original queries. This step aimed to mitigate the risk of missing seminal or domain-adjacent contributions due to vocabulary mismatches or indexing limitations. While we did not perform a full backward and forward snowballing procedure, this supplementary screening provided an additional layer of coverage beyond database search alone.

#### 2.2.2 Inclusion and exclusion criteria

The articles appearing in the resulting pool of articles are not necessarily useful in answering the research questions defined above. For this reason, most of the literature review methodologies (Brereton et al., 2007; Galster et al., 2013) apply a set of exclusion criteria to retain only pertinent articles. The set of exclusion criteria, defined by the authors, is listed below.

**–EC.1:** Invalid field: This review focuses exclusively on computer sciences. As such, papers from fields like human sciences are excluded.
**–EC.2:** No norm identification: Papers that do not directly focus on approaches to identify norms are excluded. As such, papers presenting an architecture taking into account norm identification, but not directly presenting this norm mining method, are discarded.
**–EC.3:** Invalid topic: Papers which are not about normative systems are excluded, as they are not relevant for our study.
**–EC.4:** Not a recent work: Papers that were published before 2009, i.e., with a publication year<2009, are excluded. It is assumed that the non-recent research is not up-to-date with the latest technologies.
**–EC.5:** Invalid type of paper, the document is a poster, a demo, a Ph.D thesis, or a preprint: It is assumed that a poster or a demo cannot give enough details on the contributions, as the contributed content is not enough for evaluation. The content of a Ph.D. thesis, on the other hand, is often published in separate papers. Preprints are excluded as they have not been peer-reviewed.
**–EC.6:** Duplicated paper: Papers being resubmissions of previous works, or collected twice under different names, are discarded, i.e., only one version is conserved among the included papers.
**–EC.7:** Extended paper: The paper is extended by another paper by the same authors. The contributions in the extended paper are enclosing the ones from the original paper, so that the latter is excluded.
**–EC.8:** Invalid type of paper, the paper is a survey: It is assumed that the survey papers (i.e., secondary studies) do not provide contributions directly on the norm identification approaches.

Norms are a cross-disciplinary concept spanning sociology, philosophy, law, and political science. In this review, we intentionally restricted the scope to computational approaches to norm identification in multi-agent systems, since our research questions focus on algorithmic detection, synthesis, and adaptation of norms. Social science literature offers valuable conceptual insights, but it rarely proposes operational or machine-interpretable methods for norm extraction. For this reason, we excluded surveys and secondary studies from the primary study set, while using them as contextual references to guide terminology and positioning. Although excluded from the primary study set, existing surveys were consulted to inform background understanding, terminology, and high-level positioning of this review. We note that definitions and taxonomies of norms vary substantially across sub-domains, and adopting a single survey framework risks introducing inconsistencies when applied uniformly to heterogeneous approaches.

**–EC.9:** Impossible to access the paper text: It is impossible to evaluate a paper when its text cannot be accessed (PDF download, online text, etc).

These exclusion criteria are applied to the documents in two steps. In the first coarse-grained step, the articles were only eliminated if their titles and abstracts satisfied at least one of the exclusion criteria. In the second fine-grained step, the remaining papers are screened, but this time, reading the whole body of the paper.

#### 2.2.3 Biases and disagreements

To mitigate the subjectivity of the reviewing process, certain measures were taken to overcome biases and resolve conflicts. In particular, each task of Phase 2 in Figure 1 was conducted by at least 2 reviewers. Thus, as shall be discussed later, the steps of article exclusion and inclusion (c.f., Section 2.3), and answering the research questions (c.f., Section 2.1), were undertaken by at least two reviewers for each article. A third reviewer intervened as a referee to resolve a conflict in the exclusion and inclusion step, and in the research question answering step.

### 2.3 Review process

This section gives an account of how the SLR has been conducted and discusses the results of the exclusion/inclusion step. Several searches on the three databases (IEEExplore, ACM DL, and Google Scholar) have been performed using combinations of the keywords “Norm Identification,” “Norm Mining,” “Norm Detection,” “Norm Discovery," “Artificial Intelligence,” and “Multi-Agent.” The criterion for stopping each search attempt was a series of 10 unrelated articles (based on their title and abstract). After this step, a total of 80 papers were collected. The next step is to apply the coarse-grained exclusion/inclusion step. Note that since this step screens papers based on their titles and abstracts, some exclusion criteria might be more helpful than others (e.g., EC.5 and EC.9).

**Figure 3:**
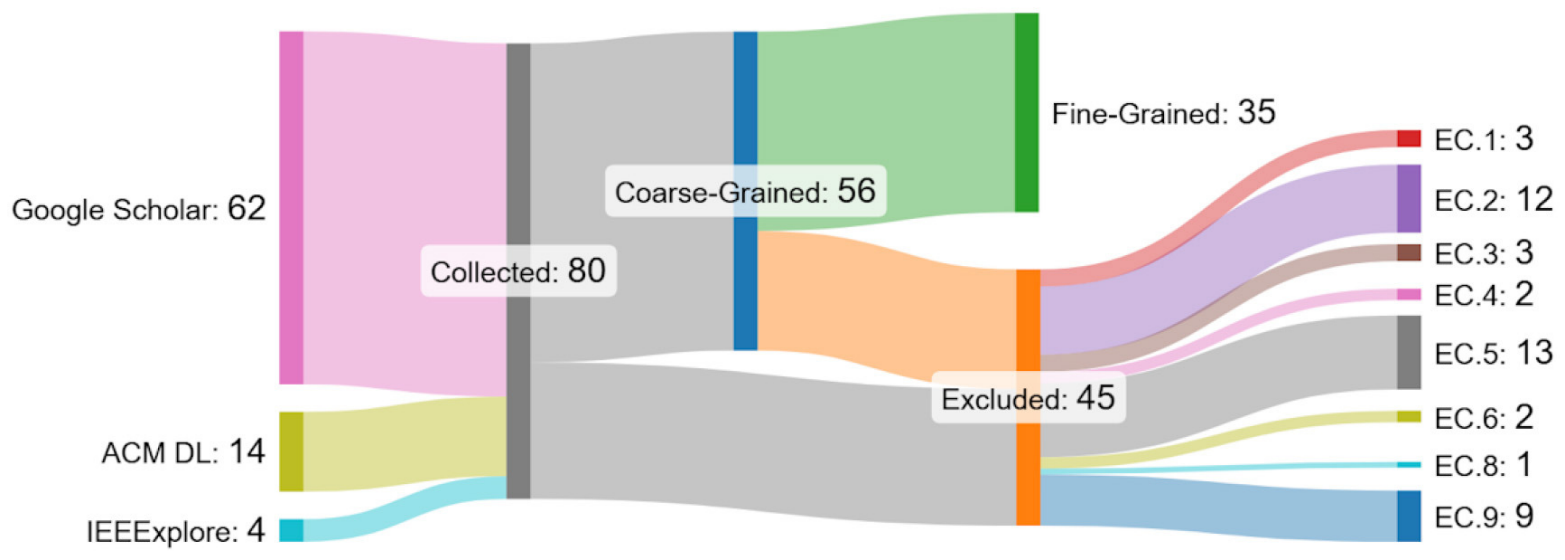
Details of the collected, included, and excluded papers.

Figure 3 presents a detailed breakdown of the number of papers at each stage of the review process, which consists of three key steps: the initial collection of papers, followed by a coarse-grained exclusion/inclusion step, and finally a fine-grained exclusion/inclusion step. The figure also highlights the most commonly applied exclusion criteria at each stage. Based on our analysis, we observe that a total of 24 papers (= 30.0%) were excluded during the first exclusion phase. An additional 21 (≈26.3%) papers were excluded in the second phase, bringing the total number of excluded papers to 45, which accounts for approximately 56.3% of the initially collected papers. Consequently, only 35 papers (≈43.7%) were ultimately included in our study.

Furthermore, it is important to note that 14 papers (= 17.5%) were subject to conflicting reviews, meaning that different reviewers provided differing assessments regarding their relevance or suitability for inclusion. In such cases, the final decision was left to the referee, who examined the discrepancies and ultimately decided to exclude 8 of these 14 papers.

One key observation from this process is that norm mining appears to be a particularly niche and narrowly focused area of research. This conclusion is supported by the fact that, despite covering a considerable time of 15 years, our extensive search efforts resulted in the identification of only 35 works that met the inclusion criteria and were considered relevant for this study.

## 3 Results and analysis

This section details some statistics and findings we could draw from the collected papers. Furthermore, it gives a more detailed overview of how each research question is answered by discussing each of them individually.

### 3.1 Demographic and temporal data

This section discusses the information collected regarding the demographic data of researchers in the field, as well as the temporal distribution of publications over the last 15 years. By analyzing publication trends, we aim to gain insights into the evolution of interest and activity in this research domain over time.

**Figure 4:**
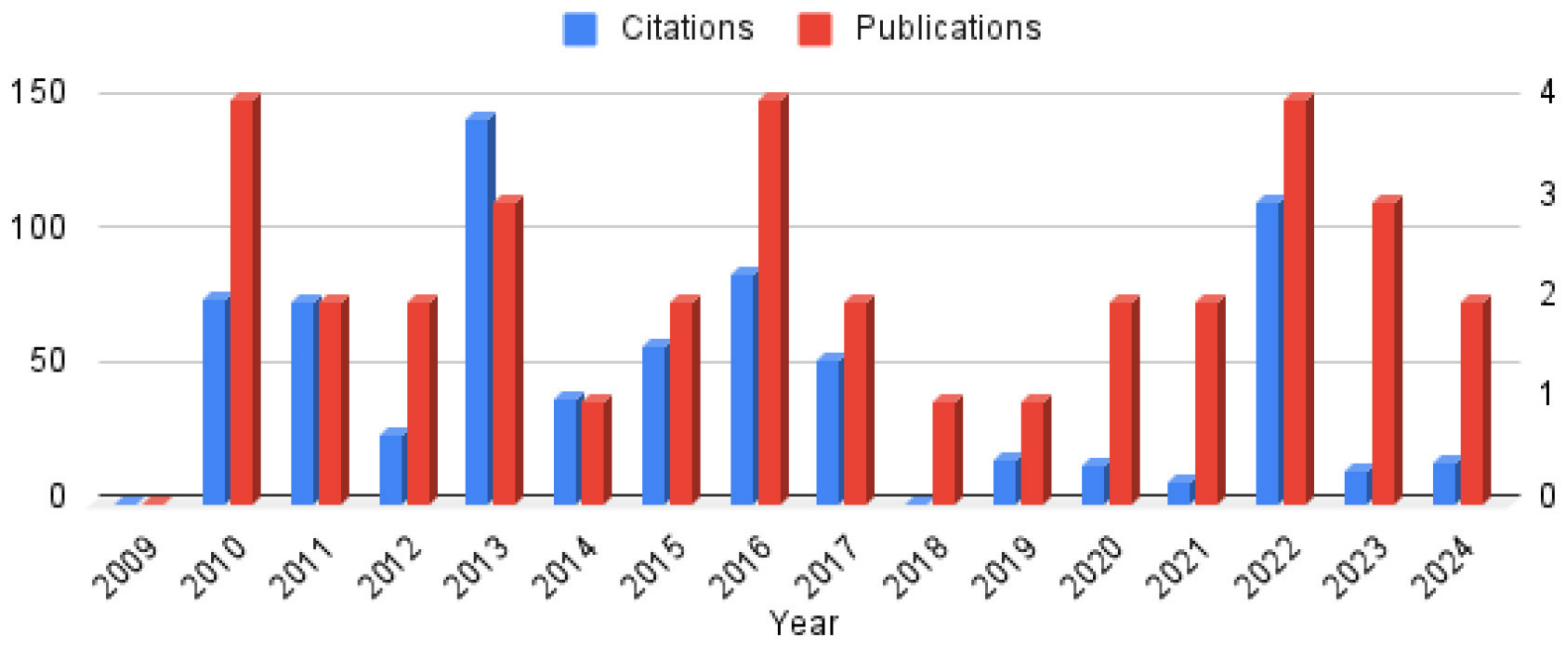
For each year, number of papers published this year (right side y-axis) and number of papers cited from this year (left side y-axis).

Figure 4 presents a dual-axis combination chart: the vertical bars (right-hand y-axis) report the number of papers published each year, while the overlaid line (left-hand y-axis) shows the cumulative citations received by papers from the corresponding year. The chart reveals three phases. From 2010 to 2013, the field experienced an exploratory phase with a modest but rising output accompanied by comparatively high citation counts. Between 2014 and 2018, publication rates stabilized at around three papers annually, while citation activity remained strong. After 2018, the number of publications remained relatively steady at one to two papers per year, indicating that research activity did not vanish. However, these more recent contributions have so far attracted far fewer citations, underscoring a decline in visibility and scholarly impact rather than in publication output. Overall, the figure highlights the persistence of research efforts in norm identification, but also a diminishing influence in terms of citation uptake.

Several factors could explain this decline. One possibility is that previous research has sufficiently addressed the fundamental challenges of norm identification, leading to a natural reduction in new contributions. We will, however, see in this paper that this is not the case and that many challenges are still present. Another potential reason is a shift in focus toward other emerging topics that are considered more relevant or pressing within the broader research community. Additionally, methodological or technological constraints may have contributed to the stagnation, discouraging further exploration.

In general, the observed trends indicate that the community studying norms is progressively losing interest in this field. This shift raises important questions about the future of norm identification research and whether renewed efforts, interdisciplinary approaches, or novel applications could help revitalize engagement in this topic.

**Figure 5:**
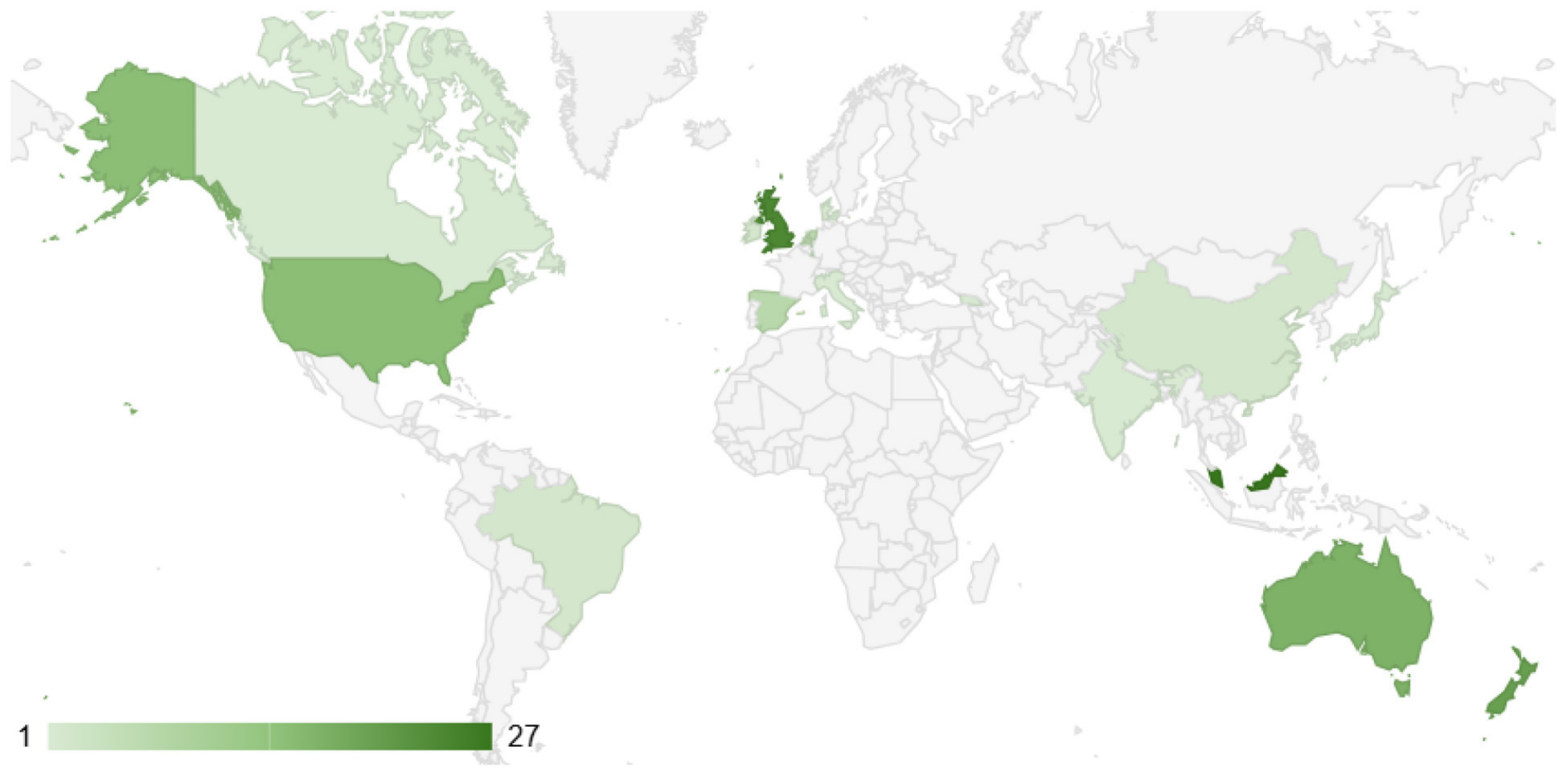
Number of times co-authors' institutions of a paper are from the designated country.

Furthermore, as shown in Figure 5, the norm identification research community appears to be quite small and geographically limited. In comparison to other research areas, even highly specialized ones, this field remains significantly underrepresented across most countries. The majority of contributions come from a handful of regions, while many countries have little to no research activity in this domain. This lack of global participation suggests that norm identification has not yet gained widespread recognition or traction within the broader academic landscape.

One possible reason for this limited presence is that norm identification may be perceived as a niche topic with fewer immediate applications compared to other well-established research areas. Additionally, the relatively small number of researchers working in this field may create challenges in terms of visibility, funding opportunities, and cross-disciplinary engagement. The absence of a strong international network can also hinder progress, as research communities thrive on collaboration, knowledge exchange, and the sharing of diverse perspectives.

For this reason, we strongly encourage researchers in this area to actively seek collaborations with colleagues from different countries. By fostering international partnerships, scholars can help expand the reach of norm identification research and stimulate interest in the field. Such collaborations could lead to the formation of new sub-communities that focus on different aspects of norm identification.

### 3.2 Analysis of the research questions

This section first describes quantitatively how the research questions were answered by the collected papers. Then, it goes more in-depth into each research question, analyzing qualitatively how the collected papers address it.

#### 3.2.1 Quantitative analysis

While each paper was addressing the challenges brought by the research questions in its own way, we could identify some major trends in the employed methods. Below are listed the keywords associated with each trend, as well as their description:

- **Threshold:** The approach observes a community of agents. If a behavior is repeated a certain number of times exceeding a threshold value, it is then added to the potential norms.
- **Comparison:** The method detects the norms by exchanging information and comparing its set of beliefs or desires with other agents from the environment, or with an external source.
- **Reasoning:** The agent uses a reasoning mechanism, or a mathematical formula, to derive the norms from the data.
- **Elitism:** The approach focuses on the observation of a limited number of agents, usually having a higher trust value. Those agents can also act as helpers toward other agents to help them in identifying the system's norms.
- **Log:** The approach makes use of the trace of other agents, potentially tracking the signals such as sanctions.
- **Data mining:** The approach uses pattern recognition techniques, or machine learning techniques, to extract the norms.
- **Natural language processing (NLP):** The method uses grammar and semantics to detect the norms.
- **Yes:** The paper answered positively to a closed research question.
- **Not answered:** The paper did not address the given research question. For a closed question, it is not necessarily equivalent to a negative answer.

**Figure 6:**
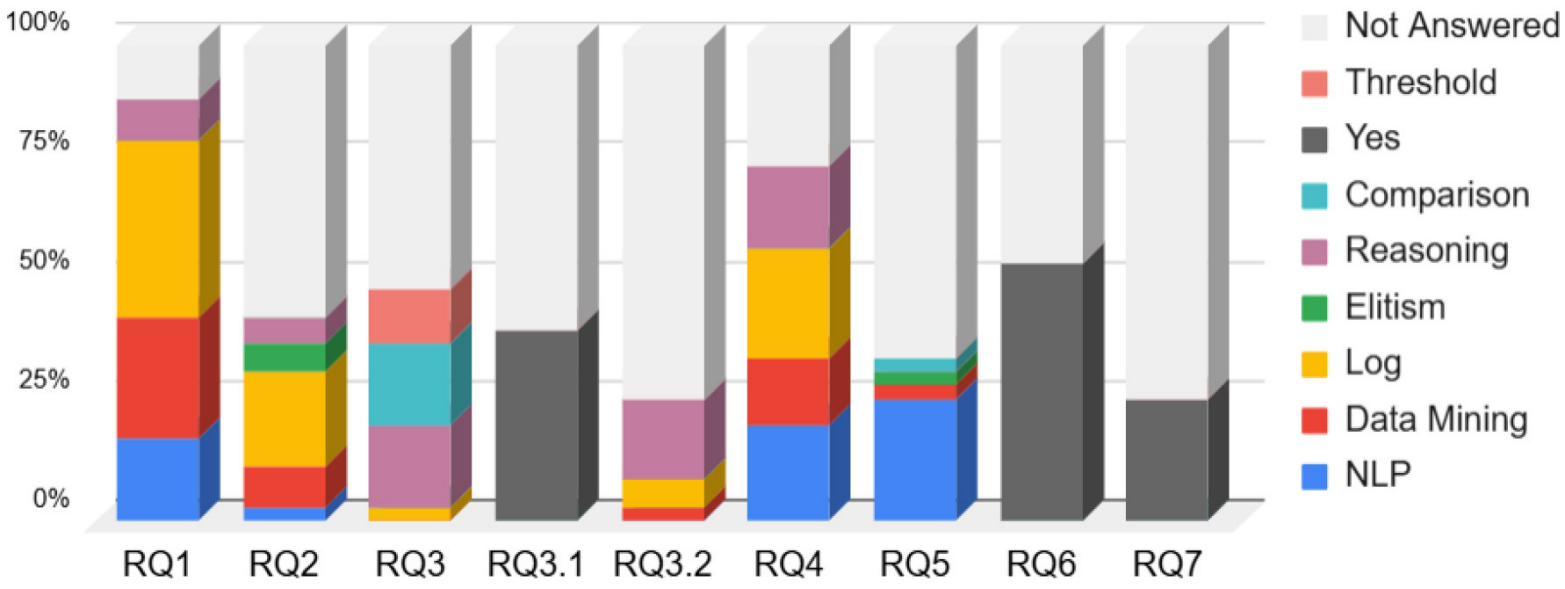
How each research question is addressed by the included papers.

Figure 6 shows, for each research question, what are the major trends among the papers answering it, as well as the proportion of papers not answering this question. Table 1 provides a detailed view of each of the collected papers.

**Table 1:** Research questions breakdown.

**No**.	**References**	**RQ1**	**RQ2**	**RQ3**	**RQ3.1**	**RQ3.2**	**RQ4**	**RQ5**	**RQ6**	**RQ7**
1	Ferraro and Lam, 2021	NLP	NLP				NLP	NLP		
2	Mahmoud et al., 2012b	Log	Log	Threshold	Yes	Data mining	Log		Yes	
3	Mahmoud et al., 2016a	Data mining		Comparison						
4	Mahmoud et al., 2012a	Log	Elitism	Comparison	Yes		Log		Yes	Yes
5	Savarimuthu et al., 2010b	Log	Log	Reasoning	Yes	Reasoning			Yes	Yes
6	Mahmoud et al., 2016b	Reasoning		Reasoning	Yes	Reasoning	Reasoning		Yes	Yes
7	Mahmoud et al., 2013	Log		Comparison		Reasoning	Reasoning		Yes	
8	Savarimuthu et al., 2010a	Log	Data mining	Comparison	Yes	Log	Log	Comparison	Yes	
9	Riad and Golpayegani, 2021	Data mining	Elitism						Yes	
10	Savarimuthu et al., 2013	Log	Log	Reasoning	Yes	Reasoning	Log		Yes	
11	Gao and Singh, 2014						NLP	NLP		
12	Cranefield et al., 2016	Data mining		Threshold	Yes				Yes	
13	Campos et al., 2010	Log	Log					Elitism	Yes	Yes
14	Avery et al., 2016	Data mining	Reasoning				Reasoning	NLP		
15	Sarathy et al., 2017	Data mining		Threshold			Data Mining		Yes	Yes
16	Dam et al., 2015		Log				Log	Data Mining		Yes
17	Aires et al., 2017	NLP					NLP			
18	Oren and Meneguzzi, 2020						Reasoning	NLP		
19	Mahmoud et al., 2012c	Data mining	Data mining	Log	Yes	Log	Data mining			
20	Murali et al., 2021	Data mining					Data mining	NLP		
21	Alechina et al., 2018	Log		Comparison	Yes		Log	NLP		Yes
22	Dell'Anna et al., 2022	Log			Yes					
23	Christelis et al., 2010	Log							Yes	
24	Morris-Martin et al., 2023	Reasoning	Reasoning	Reasoning	Yes	Reasoning			Yes	Yes
25	Morales et al., 2013	Log	Log	Comparison			Reasoning		Yes	
26	Morales et al., 2015	Reasoning	Log	Reasoning	Yes	Reasoning	Reasoning		Yes	
27	Liga and Robaldo, 2023	NLP					NLP			
28	Liga and Palmirani, 2022	NLP					NLP			
29	Corapi et al., 2011	Log		Reasoning	Yes		Log		Yes	
30	Tan et al., 2019	Data Mining		Threshold	Yes		Data Mining		Yes	
31	Cranefield and Dhiman, 2021	Log					Log			
32	Pham et al., 2024							NLP		
33	Oldenburg and Zhi-Xuan, 2024	Data mining	Data mining				Data mining		Yes	
34	Fung et al., 2023	NLP					NLP	NLP	Yes	Yes
35	Moghimifar et al., 2023	NLP					NLP	NLP		

**Figure 7:**
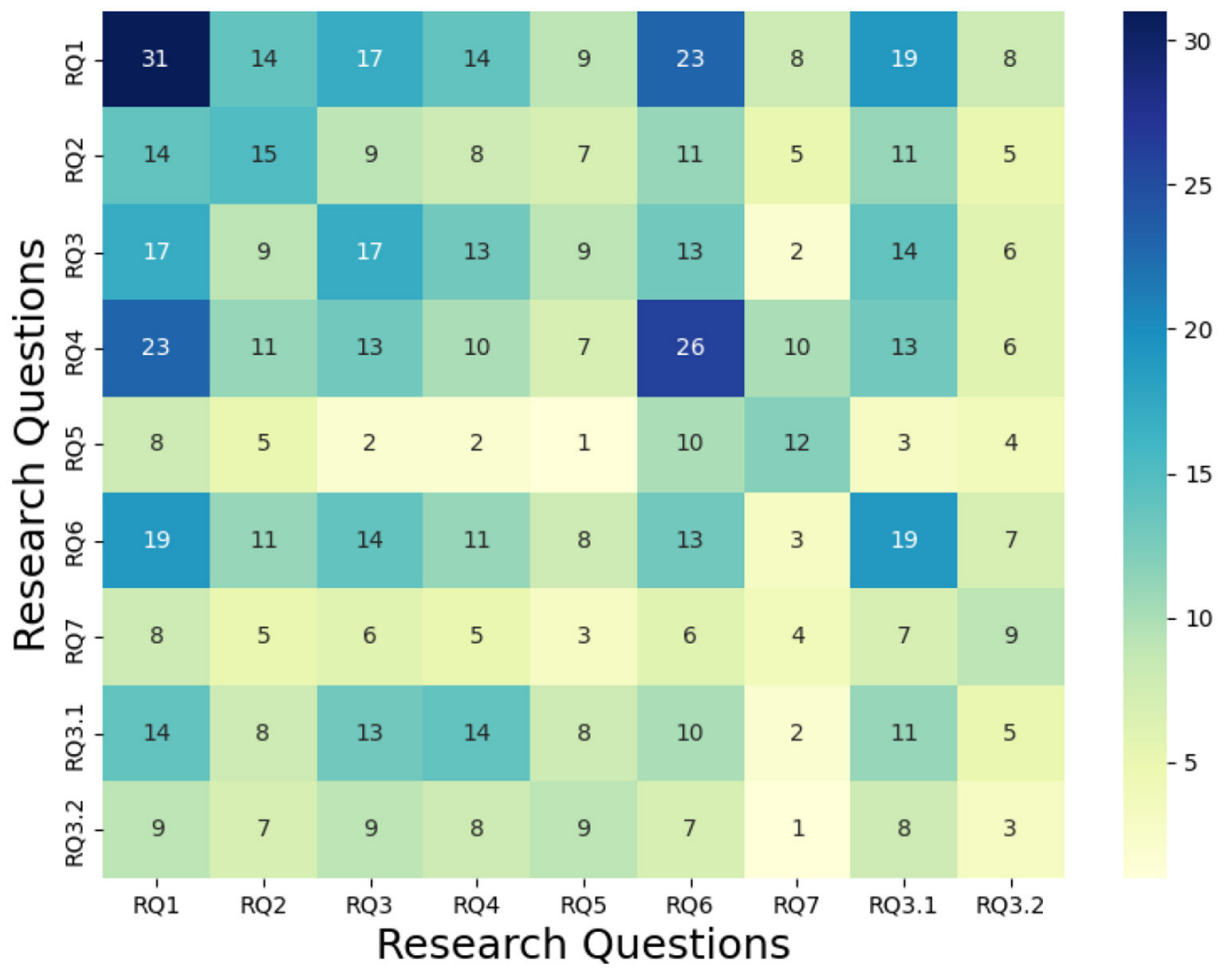
Comparison with the maximal value.

**Figure 8:**
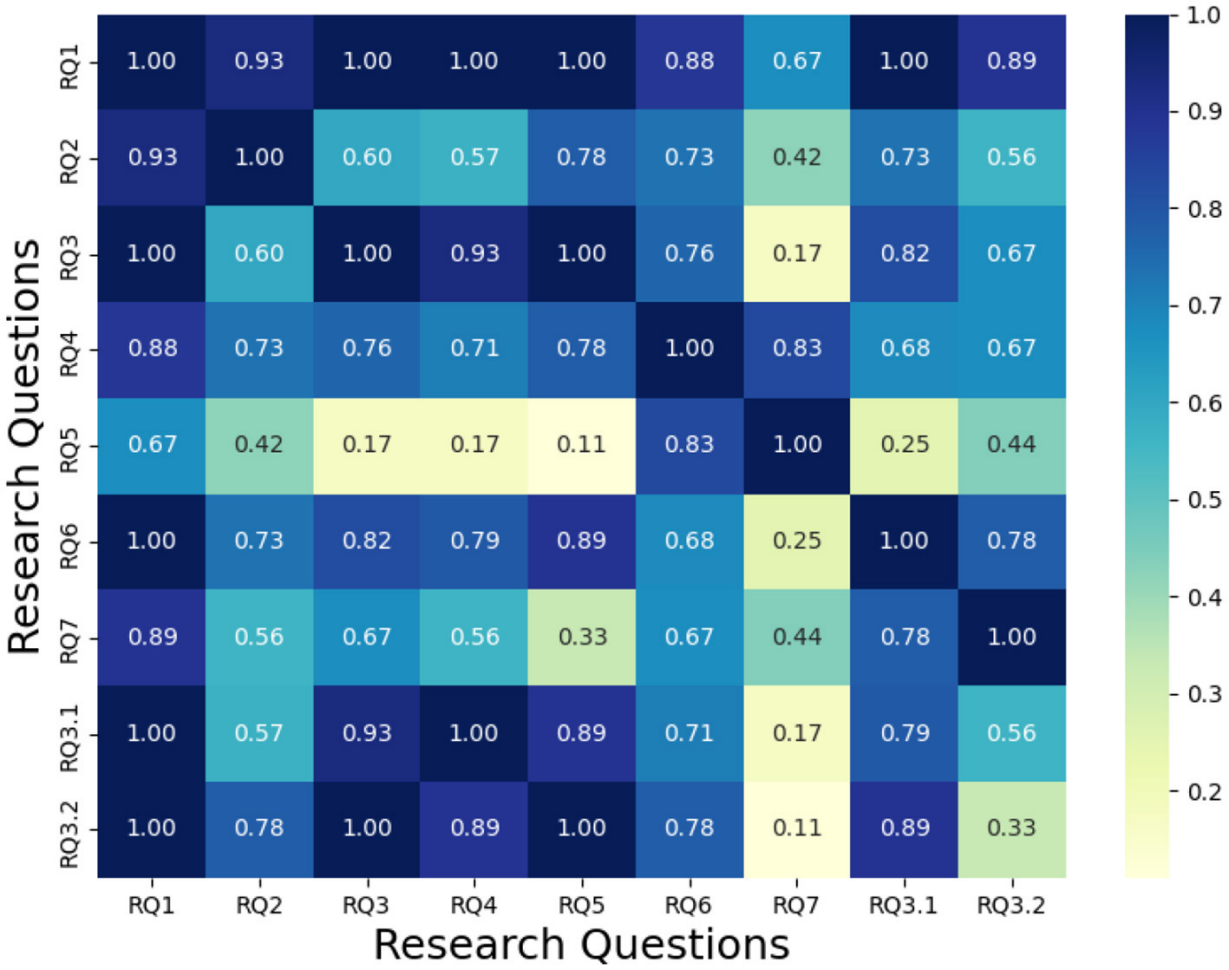
Comparison with the minimal value.

We were also interested in the correlation between the research questions, which are answered by the same papers. As such, Figure 7 shows the correlations among research questions by considering the most answered research question. On the other hand, Figure 8 shows the correlation, given the minimum value of the number of papers answering the research question between two questions.

It is important to note that papers not focusing on or taking into account MAS may be unable to answer a part of the research questions.

Furthermore, we would like to emphasize that among the collected papers, about half are exempt of implementation and empirical evidence, or are solely tested over what we could qualify as toy examples—examples or environments with discrete and rather small state and action spaces, and not using any real-world data. Out of the 35 papers, 6 (≈17.1%) do not have any experimental results, 13 (≈37.1%) use a toy example, and 16 (≈45.7%) use more sophisticated environments.

#### 3.2.2 Qualitative analysis

In the quantitative analysis, we summarized which trends appear most frequently for each research question. In this qualitative analysis, we focus on interpretability for the reader: for each research question, we (i) summarize the main methodological families, (ii) state their typical strengths and limitations, and (iii) indicate the conditions under which each family is most applicable.

**RQ.1: How to identify the norms of a society by observing it?**

Various approaches have emerged in the literature, including: Frequency-based detection methods, such as the Potential Norms Mining Algorithm (PNMA) proposed by Mahmoud et al. (2013), which identifies norms through statistical analysis of observed behavioral patterns. Bayesian hypothesis testing, as introduced by Cranefield et al. (2016), calculates the likelihood of a candidate norm's existence. Plan recognition approaches by Oren and Meneguzzi (2020) that infer norms through observed action sequences. These approaches are convenient as they allow for the extraction of norms without causing any disturbance to the system. Furthermore, they allow for a simple but comprehensive justification of why a norm was extracted by relying on its frequency of occurrence. Yet, these methods also struggle when facing a limited set of agents as they would become more sensitive to the individual actions of each agent, and thus would be prone to the extraction of personal norms rather than global norms. While deviating a bit from the research question as they do not properly observe agents, repository mining techniques (Dam et al., 2015) and legal text analysis (Ferraro and Lam, 2021) provide insight into normative structures by extracting patterns from unstructured or semi-structured documents.

*Applicability:* Observation-based approaches trade safety for ambiguity. Frequency and thresholding methods can be simple to justify and implement, but they tend to confuse individual habits with group-level regularities when the number of observed agents is small or when behavior is heterogeneous. Bayesian and probabilistic approaches provide calibrated confidence and can reduce false positives, but they rely on modeling assumptions and require enough evidence for stable posterior estimates. Plan recognition can infer structured constraints from action sequences, but it presumes a task model or plan library, which may not exist in open-ended environments. Overall, observation is most suitable when direct experimentation is undesirable, but it often needs complementary mechanisms to disambiguate personal norms from societal norms.

**RQ.2: How to identify the norms of a society by interacting with it?**

The literature presents several interaction-based approaches: Similarly, verification through peer interactions (Savarimuthu et al., 2013) to confirm candidate norms and query-based approaches (Savarimuthu et al., 2010a) that actively test for norm existence are also addressed. In contrast to the previous subsection, these approaches have the advantage of being applicable even when the system contains no agents other than the interacting one. However, they require the environment to provide a mechanism for sanctioning or notifying the agent when a violation occurs. Moreover, the set of extracted norms mainly relies on the quality of the exploration, since a norm that is never triggered cannot be inferred. This limitation can be partially addressed by combining these methods with observation techniques, such that they are not used to detect all norms, but rather to explore uncertain ones and to distinguish personal norms from global norms. Finally, these methods may themselves commit violations, which can be problematic when learning in a real-world environment rather than in a simulation.

*Applicability:* Interaction-based approaches are sample-efficient for norms that generate feedback, but they are constrained by what the environment reveals. They are well-suited to settings where sanctions, explicit notifications, or reliable feedback signals exist, since norm inference depends on observing consequences of actions. Their main weakness is coverage: norms that are rarely triggered, or that cannot be safely violated, may never be inferred. A practical pattern is to use interaction primarily for confirmation and disambiguation of uncertain candidates, while relying on observation to generate an initial pool of candidate norms.

**RQ.3: How to differentiate individual norms from societal norms in a multi-agent society?**

Mahmoud et al. (2013) and Mahmoud et al. (2016a) explore frequency-based differentiation, distinguishing descriptive norms (emerging from agent behaviors) from injunctive norms (those explicitly reinforced). Peer verification mechanisms (Savarimuthu et al., 2013) provide a means of validating societal norms vs. personal behaviors through sanction-based differentiation. **RQ.3.2** remains an open problem in the literature, as most approaches rely on multi-agent interactions. However, Bayesian updates (Cranefield et al., 2016) and agent-centered norm evaluation (Savarimuthu et al., 2010b) suggest that individual agents could infer societal norms through probabilistic reasoning and historical observation.

*Applicability:* Differentiating personal norms from societal norms requires more than frequency. Frequency-based heuristics can work when group norms are dominant and the population is large, but they break down under sub-communities, shifting populations, or strong individual strategies. Sanction-based verification provides clearer evidence of group-level enforcement, but it presumes observable sanctions and may miss norms that are followed without explicit punishment. Probabilistic methods can, in principle, represent uncertainty about whether a behavior is idiosyncratic or social, but they still require identifiable signals that separate individual preference from collective constraint. This research question remains difficult because it demands evidence about enforcement or shared expectation, not only repetition.

**RQ.4: How to detect prohibition norms without norm enforcement?**

Savarimuthu et al. (2013) and Dam et al. (2015) present data-driven approaches using association rule mining and repository analysis to identify prohibition norms, while Bayesian event sequence analysis (Murali et al., 2021) estimates prohibition likelihood based on historical compliance trends. The other approaches include analysis of infrequent patterns (Mahmoud et al., 2013) that may indicate avoided behaviors, detection of absence patterns in expected action sequences (Mahmoud et al., 2012a,c), linguistic cues in communications (Aires et al., 2017; Ferraro and Lam, 2021) that signal prohibited actions, and avoidance patterns in plan execution (Oren and Meneguzzi, 2020). In this set of approaches, the main trend seems to be to follow the assumption that the less a behavior is adopted, the more likely it is to be prohibited. However, this raises significant challenges when it comes to detecting prohibitions that have not been triggered by an agent violating them. Although it may be possible in a small environment to assume that, among all the possible actions, the ones not performed are prohibited, this is not a scalable option in larger environments.

*Applicability:* Detecting prohibitions without enforcement is fundamentally underdetermined from behavior alone. Infrequent-action heuristics can suggest candidates, but rarity is not equivalent to prohibition, especially in large action spaces where many actions are simply irrelevant. Methods that exploit structural expectations, such as plan-based models, can infer missing actions more meaningfully, but they require a task model and assumptions about rational planning. Communication and text-based cues can provide direct evidence of prohibitions, but only when such linguistic signals exist and are accessible. In practice, prohibition detection without enforcement benefits from combining multiple weak signals rather than relying on absence of behavior as a single indicator.

**RQ.5: How to detect norms in communications?**

Approaches include natural language processing of communications logs (Avery et al., 2016; Dam et al., 2015), extraction techniques specialized for formal documents like contracts (Gao and Singh, 2014), analysis of modal verbs and deontic expressions (Aires et al., 2017; Ferraro and Lam, 2021), and event analysis from communication records (Murali et al., 2021). The main limitation of these techniques is that they are usually not agnostic to the environment or application scenario as they require the knowledge of the language used by the agents. Changing this environment would require a redesign of the expressions used to detect the norms, or a new learning phase to match the newly encountered dialogue patterns.

*Applicability:* Communication-based norm detection is effective when norms are explicitly stated, hinted through deontic language, or embedded in recurring dialogue patterns. Its main limitation is domain and language dependence, since extraction rules and learned patterns may not transfer across communities, genres, or languages. Formal documents such as contracts can offer clearer structure, while free-form conversations require stronger semantic modeling and typically yield noisier outputs. These methods are most applicable when textual traces are abundant and when norms are articulated in language rather than only enacted through behavior.

**RQ.6: Is it possible to adapt to drifting norms without restarting the whole learning phase?**

Several works address norm adaptation by allowing agents to update their normative models over time rather than relearning from scratch. Riad and Golpayegani (2021) propose online norm synthesis mechanisms guided by utility-based adaptation, where norms are revised continuously in response to environmental feedback. Mahmoud et al. (2016b) introduce an assimilation-based perspective, in which agents incrementally adjust their behavior to align with evolving norms while considering the cost of integration into different normative groups.

This contribution marks a shift compared to earlier works by the same authors. While previous studies focused primarily on identifying candidate norms from observations or interactions, Mahmoud et al. (2016b) emphasize long-term integration, framing norm adaptation as a problem of joining and remaining within a normative sub-community under bounded assimilation cost, rather than only detecting what the norms are.

Other approaches to handling norm drift include continuous monitoring and incremental updates (Mahmoud et al., 2012a, 2013, 2012b), Bayesian updating mechanisms that revise confidence in norms over time (Cranefield et al., 2016), case-based reasoning for adapting norms in evolving systems (Campos et al., 2010), and online refinement techniques for dynamic norm synthesis (Morales et al., 2013, 2015). Together, these methods support sustained operation in changing environments, complementing norm mining techniques that mainly address initial integration.

A recurring limitation across these approaches is their limited consideration of population dynamics. When multiple agents using similar learning architectures are introduced over time, mutual adaptation may dampen exploration. In frequency-based methods in particular, agents can converge toward each other's early behavior, creating inertia that slows or prevents adaptation to newly emerging norms.

*Applicability:* Approaches to norm drift differ mainly in what they treat as evidence of change. Incremental update and monitoring methods can track gradual shifts, but they can be slow to react to abrupt regime changes. Bayesian updating provides principled confidence adjustment, but it depends on a stable likelihood model and can lag when the data distribution changes sharply. Utility-driven and synthesis-based methods can revise norms more actively, but they require explicit objective modeling and can introduce instability if revisions are too frequent. A practical open issue is population churn: if many agents with similar learning mechanisms enter over time, their mutual adaptation may dampen exploration and slow down the discovery of new norms.

**RQ.7: Is it possible to detect sub-communities of agents?**

Campos et al. (2010) and Morris-Martin et al. (2023) discuss case-based reasoning and agent-directed norm synthesis as potential solutions. These methods offer promising avenues for sub-community detection but also introduce challenges related to scalability and the granularity of norm differentiation within and across sub-communities. Mahmoud et al. (2012a) analyze behavioral clustering to identify societal subdivisions while other approaches include comparative analysis of repositories (Dam et al., 2015) to identify community-specific norms, movement-based detection (Savarimuthu et al., 2010b) that analyzes agent groupings, and analysis of heterogeneous groups (Mahmoud et al., 2016b) with distinct normative systems. The critical element in these approaches often lies in finding the right balance such that sub-communities are identified but personal norms remain excluded from the norm detection process. If done successfully, not only it allows these approaches to identify the norms of a system effectively, but they also become resilient to personal norms and noise in the environment.

*Applicability:* Sub-community detection sits between clustering and norm inference. Clustering agents by behavior can reveal groups, but it does not guarantee that the separating features correspond to normative constraints rather than preferences or roles. Repository and trace comparisons can highlight group-specific regularities, but they require comparable logs and may be sensitive to missing data. The key technical difficulty is choosing a granularity where group structure is detected while individual outliers do not dominate the inferred norms. This is also why results from RQ3 are relevant here: methods that separate personal from group norms can be repurposed as a building block for sub-community discovery.

### 3.3 Analysis of the collected papers

This section presents a classification of the context in which each proposed approach operates, as well as a comprehensive review of the included papers.

#### 3.3.1 Classification of the reviewed approaches

After reviewing the collected papers, we identified two major categories based on their application context: *Agent-Based* and *Not Agent-Based* approaches. A method is considered Agent-Based if it identifies norms through the interactions of agents within an environment or through their communication with other individuals. The key characteristic of these approaches is the presence of actions (i.e., interactions) that facilitate the discovery of norms. In contrast, a method is classified as Not Agent-Based if it primarily relies on data analysis rather than interactive behaviors. Each of these categories can be further divided into subcategories.

Agent-Based approaches can be grouped into three subcategories (Oren and Meneguzzi, 2020): *Observatory, Experiential*, and *Communicative*. Observatory approaches rely on observing other agents (or traces of their actions) interacting in an environment. These methods are considered safe since they do not involve direct experimentation that could lead to norm violations. However, they may struggle to identify prohibitions, particularly when all observed agents comply with existing norms, leaving no violations to be detected. Experiential approaches operate on trial and error. This method is commonly found in behavior learning techniques such as Reinforcement Learning. It is typically efficient and relatively simple to implement. However, unlike Observatory methods, it involves committing multiple norm violations before correctly identifying the normative behavior. Communicative approaches rely on exchanging information with already integrated agents. Like Observatory methods, they are relatively safe. However, they tend to be the most complex to implement effectively, which limits their practical use.

**Figure 9:**
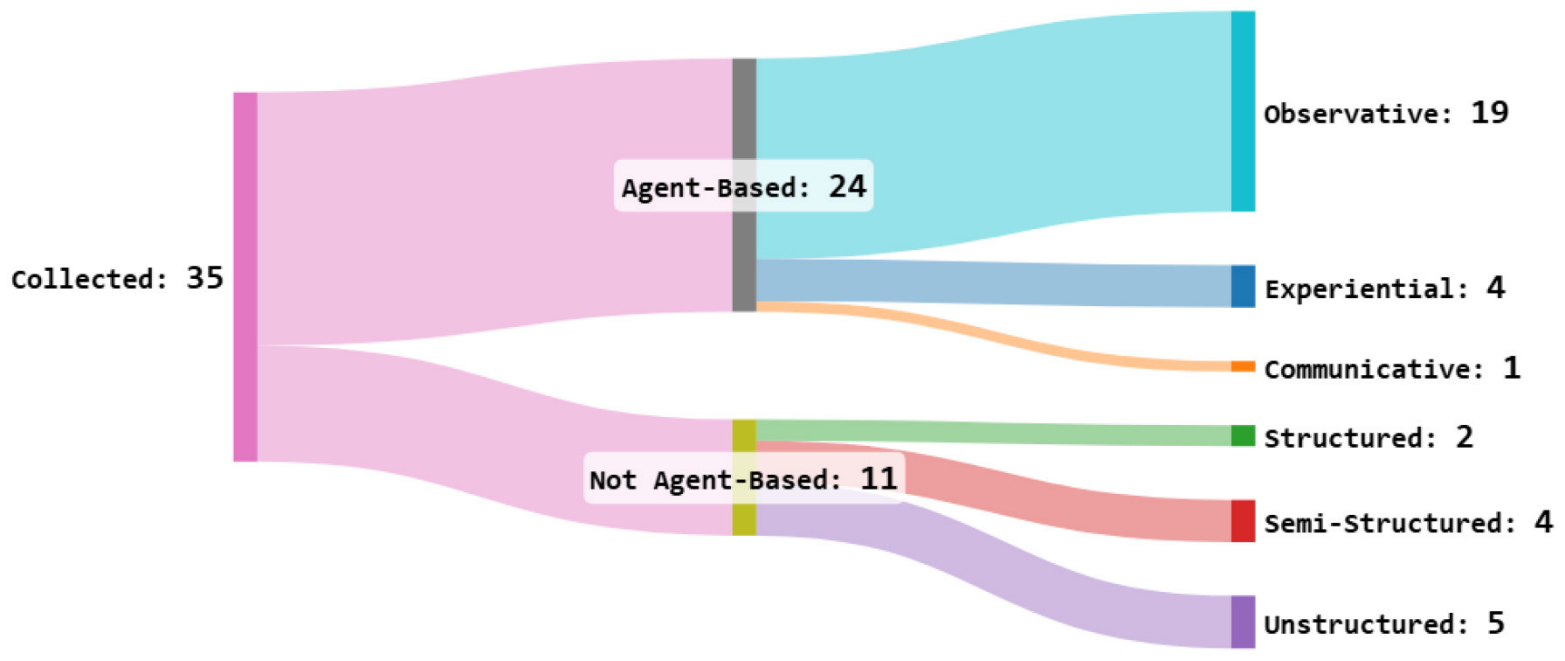
Taxonomy of the approaches proposed in the collected papers.

Methods that do not fall under the Agent-Based category are distinguished by the type of data they process. We identified three subcategories: *Structured, Semi-Structured*, and *Unstructured* data. Structured data consists of pre-encoded information, such as databases, where symbolic elements are already extracted and standardized. Approaches in this category typically apply pattern recognition techniques to identify norms. Semi-structured data includes documents that follow a standardized structure and contain recognizable keywords related to norms. Examples include legal texts and contracts. These approaches are more challenging than Structured Data methods but remain more manageable than Unstructured Data methods. Unstructured data encompasses free-form content, such as forum discussions and natural language documents. Because these data sources are unprocessed, extracting meaningful symbols for norm identification is significantly more complex. Among the collected approaches, none addressed audio or video data, even though these are potential media for norm identification. This classification is illustrated by Figure 9.

**Figure 10:**
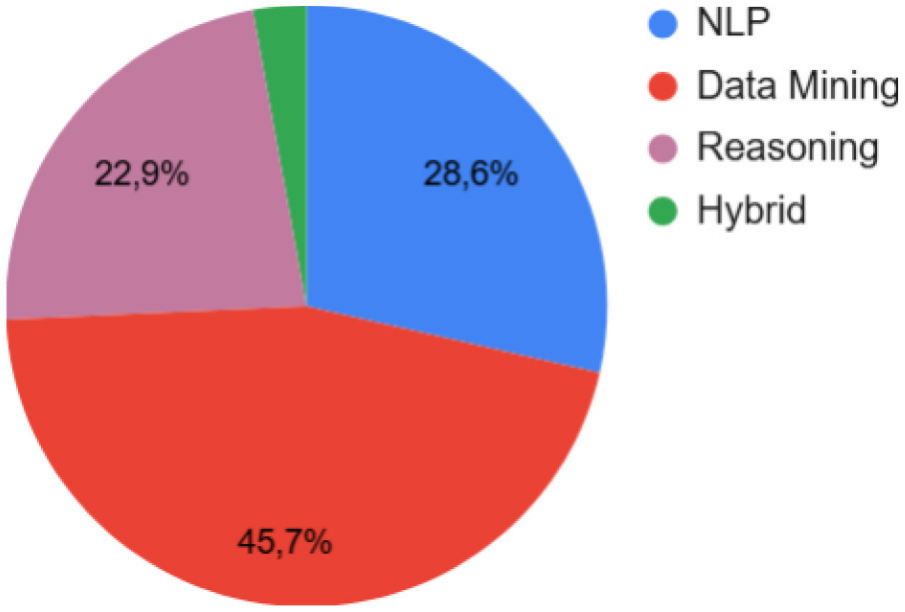
Main area of the approaches proposed among the collected papers.

In addition to the taxonomy based on methodology, we also classified the collected papers according to their primary research focus, see Figure 10. We identified three main areas: *Natural Language Processing (NLP), Data Mining*, and *Reasoning*. Additionally, we introduced a *Hybrid* category for approaches combining at least two of these areas. NLP approaches focus on the semantic analysis of textual data to extract norms. Data Mining approaches analyze large datasets to identify patterns and infer norms. Reasoning approaches derive conclusions from limited data and refine their findings as more information becomes available. Hybrid approaches integrate elements from multiple research areas to enhance norm identification. Figures 9, 10 illustrate the distribution of papers across these categories.

#### 3.3.2 Norm detection and identification

Several researchers have investigated techniques for detecting and identifying norms in MAS. Mahmoud et al. (2013) propose a Potential Norms Mining Algorithm (PNMA) that enables agents to identify prevailing norms through observation of other agents' behaviors. Their approach allows an agent to revise its norms without requiring third-party enforcement mechanisms. The PNMA follows a structured process of data formatting, filtering, and extraction of potential norms from observed events. Building on this work, Mahmoud et al. (2016a) present the Potential Norms Detection Technique (PNDT), which facilitates agents' adaptation to changing environments through self-enforcement. The PNDT framework comprises an agent's belief base, observation process, the PNMA algorithm, verification process, and updating process. Through simulations in an elevator scenario, they demonstrate how environmental variables affect norm detection success. Cranefield et al. (2016) introduce a novel approach using Bayesian inference for norm identification. Their method effectively operates in scenarios where both compliance and violation occur regularly, calculating the odds of a candidate norm being established vs. no norm existing. Empirical evaluation shows that norm-compliant behavior can emerge after relatively few observations. Oren and Meneguzzi (2020) develop a norm identification mechanism based on plan recognition, combining parsing-based plan recognition with Hierarchical Task Network planning to infer prevailing norms. Their approach handles norm violations through counting and thresholding, without relying on observation of explicit sanctions. Sarathy et al. (2017) propose a norm representation scheme incorporating context-specificity and uncertainty using Dempster-Shafer theory. Their algorithm learns norms from observation while considering different contexts and the inherent uncertainty in the learning process, allowing agents to adapt to changing contexts.

#### 3.3.3 Norm mining from data

Several researchers have explored data mining techniques for extracting norms from various sources. Savarimuthu et al. (2010a) present an internal agent architecture for norm identification based on interaction observation. Their Obligation Norm Inference algorithm uses association rule mining to identify obligation norms. In related work, Savarimuthu et al. (2013) focus on identifying prohibition norms using a modified version of the WINEPI algorithm to generate candidate prohibition norms. Their framework considers social learning theory and distinguishes between candidate norms and identified norms. Savarimuthu et al. (2010b) further develop their architecture with the Candidate Norm Inference algorithm, which identifies sequences of events as candidate norms. Their approach enables agents to modify and remove norms if they change or no longer hold in the society, demonstrating the benefits of norm inference for utility maximization. Avery et al. (2016) introduce Norms Miner, a tool for extracting norms from open source software development bug reports. Their automated approach discovers, extracts, and classifies norms from textual social interactions, making tacit knowledge explicit and accessible. The tool achieves solid performance with a recall of 0.74 and a precision of 0.73 in norm classification.

Dam et al. (2015) explore mining software repositories for social norms, presenting results on coding convention violations across large open source projects. They propose a life-cycle model for norms within Open Source Software Development communities and demonstrate its applicability using data from the Python development community. Ferraro and Lam (2021) apply Natural Language Processing techniques to normative mining from legal documents. They provide a comprehensive review of existing NLP techniques, particularly semantic parsing, and analyze their applicability to mining legal norms. The paper presents preliminary results on extracting normative rules using relation extraction and semantic parsing models. Gao and Singh (2014) develop an approach for automatically extracting norms from contract text. Their prototype tool suite extracts norms and related concepts, evaluating the realism of normative models in MAS by assessing how effectively these concepts can be identified within contracts. Murali et al. (2021) apply norm-mining techniques to a real-world dataset in international politics. They adapt a Bayesian norm mining mechanism to identify norms from bilateral sequences of inter-country events extracted from the GDELT database, demonstrating that a model combining probabilities and norms explains observed international events better than a purely probabilistic model.

#### 3.3.4 Norm assimilation and adaptation

Several researchers have explored how agents can assimilate and adapt to norms in MAS. Mahmoud et al. (2012b) propose a technique for software agents to detect and assimilate norms to comply with local normative protocols. Their conceptual framework includes stages for a visitor agent to detect norms by analyzing interaction patterns and matching them with a “norms model base.” Mahmoud et al. (2016b) introduce a norm assimilation approach for MAS in heterogeneous communities. Their theoretical framework is based on an agent's internal belief about its ability to assimilate and its external belief about the assimilation cost associated with different social groups. They categorize assimilation decisions based on whether an agent “can assimilate,” “could assimilate,” or “cannot assimilate.” Mahmoud et al. (2012c) focus on defining the semantics of a proposed norms mining technique. They explicitly define the semantics of the entities and processes involved in norms mining, drawing inspiration from existing work in norms, normative systems, and data mining. Mahmoud et al. (2012a) outline a conceptual approach for norms detection and assimilation, focusing on discovering norm emergence based on interaction patterns between agents. Their approach utilizes a norms mining technique and proposes using a norms learning technique to define the semantics of textual data.

#### 3.3.5 Norm synthesis and revision

Several researchers have investigated techniques for synthesizing and revising norms in MAS. Morales et al. (2013) introduce IRON (Intelligent Robust On-line Norm synthesis mechanism), which synthesizes conflict-free norms without over-regulation. IRON produces norms that characterize necessary conditions for coordination and are both effective and necessary, with the capability to generalize norms for concise normative systems. Morales et al. (2015) present an extended IRON mechanism designed for online synthesis of compact normative systems. Their enhanced approach incorporates improved evaluation methods, a generalization operator requiring sufficient evidence, and a specialization operator for refining underperforming generalizations. Empirical evaluation shows that IRON significantly outperforms BASE in terms of stability and compactness. Riad and Golpayegani (2021) propose a utility-based norm synthesis model for managing norms in complex MAS with multiple, potentially conflicting objectives. Their approach employs utility-based case-based reasoning for run-time norm synthesis, using a utility function derived from system and agent objectives to guide norm adoption. Dell'Anna et al. (2022) analyze the complexity of synthesizing and revising conditional norms with deadlines. They demonstrate that synthesizing a single conditional norm correctly classifying behavioral traces is NP-complete, as is synthesizing sets of conditional norms and minimal norm revision. Christelis et al. (2010) detail a first-order approach to norm synthesis, allowing for greater expressiveness through the use of variables. They propose optimizations to improve the performance of first-order norm synthesis, including a priori filtering, traversal pruning, repetitive operators, and duplicate runs. Morris-Martin et al. (2023) propose an agent-directed norm synthesis framework that allows norms to be synthesized based on agent requests and interactions. Their approach involves individual agents in system governance, enabling revisions that benefit individual goals without conflicting with system-level objectives.

#### 3.3.6 Norm conflict detection and resolution

Aires et al. (2017) focus on identifying potential conflicts between norms in contracts written in natural language. They develop a semi-automatic approach for identifying norms and their elements using information extraction techniques. Their tool assists in preventing conflicts by comparing extracted norm information and classifying potential conflicts into types like permission-prohibition, permission-obligation, and obligation-prohibition. Alechina et al. (2018) address the problem of detecting norm violations in open MAS. They demonstrate that perfect or near-perfect norm monitoring and enforcement can be achieved at no cost to the system, proposing incentive-compatible mechanisms for decentralized norm monitoring where agents themselves perform monitoring. Campos et al. (2010) propose adding an “Assistance layer” to MAS to handle norm adaptation. They use a Case-Based Reasoning approach within this layer, enabling the system to learn from past experiences and adapt norms to achieve organizational goals, illustrated through a Peer-to-Peer sharing network scenario.

### 3.4 Discussion

This paper presents a systematic review of norm detection, mining, and adaptation techniques. The analysis reveals significant progress in addressing key challenges, particularly in norm identification through passive observation (RQ1) and active interaction (RQ2). Approaches such as Bayesian inference, plan recognition, and NLP-driven mining demonstrate robust methodologies for extracting norms from behavioral traces, legal texts, and communication logs. However, critical gaps remain.

The differentiation of individual and societal norms (RQ3) remains underdeveloped, with most methods relying on multi-agent interactions or frequency-based heuristics. Sub-questions RQ3.1 and RQ3.2, which probe the feasibility of distinguishing personal norms from group norms using limited data, are notably underexplored. While probabilistic reasoning and sanction-based verification offer partial solutions, a unified framework for norm differentiation in decentralized settings is absent.

Prohibition norm detection (RQ4) and norm extraction from communications (RQ5) benefit from advances in data mining and NLP, yet these techniques often depend on structured datasets or explicit linguistic markers, limiting their applicability to noisy, real-world environments. Norm adaptation (RQ6) emerges as a well-studied area, with online synthesis and incremental updates showing promise for handling norm drift. In contrast, sub-community detection (RQ7) lacks scalable solutions, as current methods focus on coarse-grained behavioral clustering or repository analysis.

The reviewed works highlight a reliance on simulation-based validation, raising concerns about generalizability. For instance, elevator scenarios and synthetic datasets dominate empirical evaluations, leaving open questions about performance in dynamic, large-scale systems. Future research should prioritize hybrid approaches—combining Bayesian methods with symbolic reasoning, or integrating NLP with multi-agent reinforcement learning—to address these limitations. Additionally, fostering interdisciplinary collaboration could bridge gaps between norm synthesis, conflict resolution, and real-world applications such as autonomous systems or legal AI.

The bar chart in Figure 6 illustrates the number of papers addressing each research question (RQ). RQ1 (“Identifying the norms by observing”) exhibits the highest coverage, reflecting its prominence in the literature. In contrast, RQ7 (“Detecting sub-communities of agents”) is the least explored, underscoring gaps in understanding norm emergence in decentralized systems. However, it is to put in relief with the papers answering RQ3.1/RQ3.2 (both sub-questions about making the distinction between group norms and personal norms), which are more answered and could potentially be adapted to address the problem of RQ7. Still, this aligns with the conclusion's critique of over-reliance on multi-agent heuristics for norm differentiation. The heatmaps in Figures 7, 8 quantify overlaps between RQs based on shared papers. Darker cells indicate stronger connections. While RQ1 overlaps with most of the other RQs, it is worth noting that answering it also incentivizes answering RQ2, RQ3, and RQ4. This can be explained by the fact that those RQs focus on the identification of norms within MAS. They also share a similar context of only detecting the norms at a given time, but not considering the evolution of those norms over time. Furthermore, RQ2 (“Identifying via interactions”) and RQ6 (“Adapting to drifting norms”) are showing a correlation higher than the average. Having a look at Figure 6, we can see that most of the approaches answering RQ2 rely on log analysis. This lets us think that most of the “interacting” approaches are using learning techniques which, once the learning phase is over, struggle with adapting to environmental changes. This correlation repeats between RQ2 and RQ4 (“Detecting norm without norm enforcement”). This is interesting as it shows that among the approaches that discover norms via interactions within the environments, many attempt to avoid committing violations during the learning process. Similarly, RQ4 and RQ5 (“Detecting norms via communications”) exhibit strong overlap, highlighting the reliance on communications of the approaches evolving in environments without explicit norm enforcement mechanisms.

Last, having a look at the taxonomy in Figure 9, we can see that a large majority of the approaches are focusing on agents and MAS. Among those approaches, most rely on observations rather than experimentation or communication. The preference for observatory methods over experiential ones can be explained by the fact that the latter may require violating the norms to learn from their actions. However, violating the norms often impacts more than just a single agent, making it a depreciated side effect. The preference for observatory methods rather than communicative methods, on the other hand, may be due to the overall complexity of the implementation of a communication protocol among agents being at the same time flexible enough to incorporate potential future norms, and simple enough so that heterogeneous agents can use it and communicate together.

## 4 Challenges and research directions

From this literature review, we were able to identify the major challenges the community of norm identification is facing, as well as some research directions addressing underexplored areas. The following list provides a synthesis of these challenges and research directions.

**RD.1:** *Adapting to drifting norms*. As normative agents are often meant to stay in an environment for a long time, rather than just accomplishing a task once, it is important to consider systems in which norms may evolve or change. Because of this, agents taking part in such systems should be able to adapt to it by either permanently revising their set of norms or detecting when a norm drift occurs to react to this change.
**RD.2:** *Detecting sub-communities*. While an important part of the work addressing problems related to MAS also answers the questions related to the differentiation between the personal and group norms, only a few extend to the problem of detecting sub-communities with possibly overlapping but distinct rules. This is, however, a problem that should be addressed as systems with heterogeneous agent types may showcase a similar structure with groups of agents sharing a different set of norms, without it being necessarily personal norms.
**RD.3:** *Establishing a communication protocol for norms*. As said earlier, having a communication protocol to allow agents to exchange information about ongoing norms can be difficult to effectively implement. Yet, we believe such a protocol could be highly valuable, as it could benefit from the same pros as observatory methods, while enabling more possibilities in particularly when dealing with heterogeneous agents or small communities.
**RD.4:** *Detecting norms in text data*. Some works explored the problem of detecting the norms in text data. However, those works often do not mention their usefulness in MAS. Yet, they would be valuable in such a context, as sometimes rules (which imply norms) can be expressed as text. Furthermore, being able to understand norms in structured and semi-structured textual data could enable the possibility for the agent to communicate about norms without the need for having a standardized language, thus making the creation of a communication protocol at the same time easier and more flexible.
**RD.5:** *Developing reasoning techniques*. While Data Mining techniques are often efficient at detecting the patterns present in the data, they may encounter difficulties in foreshadowing the potential other norms that were not highlighted by the data. We believe that reasoning, because of its inference capabilities, could supplement data mining approaches by deriving additional norms from the ones derived by the data.
**RD.6:** *Enlarging the scope of norm identification to more than MAS*. As shown by some of the collected papers, norms are present in a broader context than just MAS. However, the works covering those other application domains are, at the moment, very few.
**RD.7:** *Testing in more complex environments*. One element we noticed while reviewing the papers was that half of them had no experiment, or were tested in small environments or toy examples. We think it is important for the field of norm identification to gain interest from the other communities, to start experimenting in more complicated scenarios, possibly getting rid of environments with a set of discrete actions. On the other hand, papers not focusing on MAS were often experimented on real-world data, like contracts, thus ensuring their correct functioning in real-world situations.
**RD.8:** *Making use of Large Language Models*. Recent advances in the field of NLP, with the arrival of Large Language Models (LLMs), have greatly simplified working with unstructured data. Unfortunately, only two works so far (Liga and Robaldo, 2023; Liga and Palmirani, 2022) have been using those models for norm mining. We believe using them could significantly improve the capacity to handle semi-structured and unstructured textual data. Furthermore, we also believe that their use could greatly simplify the process of creating communicative approaches, as heterogeneous agents would then be able to communicate about norms.

## 5 Conclusions

Norm identification is essential for ensuring coordinated behavior in multi-agent systems. This review highlights the different methods used, their strengths, and their limitations. While rule-based and data-driven approaches offer useful insights, they struggle with scalability and adaptability in dynamic environments. Hybrid methods show promise but require further refinement. Our study identifies key research gaps, such as improving real-time norm adaptation and handling uncertainty. Future work must prioritize hybrid methods (e.g., combining reasoning with machine learning), real-world testing, and leveraging LLMs for unstructured data. Interdisciplinary collaboration and scalable solutions for sub-community detection are critical to enable autonomous agents to operate seamlessly in dynamic human environments. We believe that more collaborations with social scientists, ethicists, and linguists could benefit the field of norm identification. Addressing these challenges will help build more reliable and intelligent multi-agent systems.

### 5.1 A roadmap for the future of norm identification

This roadmap serves to describe how the research on norm identification can grow and improve over time. Although norm identification is an important topic for building social AI systems, it has received little attention in recent years. To move the field forward, we propose three main phases. These phases start with building better tools and methods and then move toward advanced, real-world applications.

#### 5.1.1 Phase I: foundational consolidation and infrastructure

The first phase addresses the current lack of standardized methods, shared experimental platforms, and accessible datasets. These limitations hinder the ability to compare and reproduce norm identification approaches. To support systematic development, we recommend the following actions:

- **Benchmark platforms:** Establish publicly accessible MAS environments where norm emergence, violation, and adaptation can be reliably simulated and evaluated.
- **Standardized evaluation frameworks:** Define performance metrics such as norm accuracy, adaptability, compliance efficiency, and behavioral explainability to ensure consistent comparison.
- **Foundational communication protocols:** Introduce simple and extensible norm-sharing protocols that allow agents to communicate their learned or inferred norms with others.

These foundational efforts will lower the entry barrier for researchers and support cumulative scientific progress.

#### 5.1.2 Phase II: methodological diversification and semantic depth

With core infrastructure in place, the second phase focuses on expanding the range of technical approaches and improving the semantic understanding of norms. This stage enables agents to operate in complex, evolving, and heterogeneous environments.

- **Lifelong norm learning:** Implement online and continual learning systems that adapt to changing environments and handle norm drift without resetting.
- **Sub-societal norm discovery:** Develop algorithms to detect sub-communities with distinct norms, using unsupervised or semi-supervised learning techniques.
- **Neuro-symbolic integration:** Combine deep learning and symbolic reasoning to support both pattern recognition and interpretability.
- **Adaptive norm inference architectures:** Create agents that can autonomously synthesize, validate, and revise norms based on structured experience and multi-agent feedback.

This phase supports the development of normative agents that are flexible, explainable, and capable of functioning in decentralized systems.

#### 5.1.3 Phase III: generalization, alignment, and societal integration

The final phase sees norm identification as a key part of general AI systems, applied in real-world settings with social, legal, and ethical dimensions. Technical development must be combined with interdisciplinary input.

- **General-purpose norm reasoning engines:** Design modular engines that work across different domains, capable of extracting and reasoning about norms in various settings.
- **Cross-cultural and legal norm corpora:** Collaborate with legal experts and ethicists to create annotated datasets of norms from laws, policies, and cultural sources.
- **Human-AI norm alignment:** Ensure agent behaviors align with ethical standards and human values by integrating sociological models and fairness principles.
- **LLM-augmented norm abstraction:** Use large language models for zero-shot norm recognition, multilingual norm explanation, and natural-language dialogue on normative topics.
- **Policy-aware agent deployment:** Apply norm-aware agents in domains like autonomous driving, digital governance, and decentralized systems, ensuring compliance with policy and regulation.

Together, these actions support the long-term goal of building intelligent agents that are not only effective but also socially responsible and legally compliant.
